# The N-Terminal Region of the Transcription Factor E2F1 Contains a Novel Transactivation Domain and Recruits General Transcription Factor GTF2H2

**DOI:** 10.3390/biom14111357

**Published:** 2024-10-25

**Authors:** Lin Zhao, Rinka Nakajima, Yaxuan Zhou, Mashiro Shirasawa, Mariana Fikriyanti, Yuki Kamiya, Hiroyuki Toh, Hideyuki Komori, Ritsuko Iwanaga, Andrew P. Bradford, Hideo Nishitani, Kenta Kurayoshi, Keigo Araki, Kiyoshi Ohtani

**Affiliations:** 1 Department of Biomedical Sciences, School of Biological and Environmental Sciences, Kwansei Gakuin University, 1 Gakuen Uegahara, Sanda 669-1330, Hyogo, Japan; ght57978@kwansei.ac.jp (L.Z.); hnj51097@kwansei.ac.jp (R.N.); gtk53096@kwansei.ac.jp (Y.Z.); idl05439@kwansei.ac.jp (M.S.); hsj19688@kwansei.ac.jp (M.F.); y.kamiya0324@gmail.com (Y.K.); tohhir@kwansei.ac.jp (H.T.); 2 Life Sciences Institute, University of Michigan, 210 Washtenaw Avenue, Ann Arbor, MI 48109, USA; hkomori@umich.edu; 3 Department of Obstetrics and Gynecology, University of Colorado School of Medicine, Anschutz Medical Campus, 12700 East 19th Avenue, Aurora, CO 80045, USA; ritsuko.iwanaga@cuanschutz.edu (R.I.); andy.bradford@cuanschutz.edu (A.P.B.); 4 Graduate School of Life Science, University of Hyogo, Kamigori, Ako-gun 678-1297, Hyogo, Japan; hideon@sci.u-hyogo.ac.jp; 5 Division of Molecular Genetics, Cancer Research Institute, Kanazawa University, Kakuma-machi, Kanazawa 920-1192, Ishikawa, Japan; kuraken0901@gmail.com; 6 Department of Morphological Biology, Ohu University School of Dentistry, 31-1 Misumido Tomitamachi, Koriyama 963-8611, Fukushima, Japan; k-araki@den.ohu-u.ac.jp

**Keywords:** E2F1, pRB, ARF, p53, transactivation domain, GTF2H2, tumor suppressor gene, cell death

## Abstract

The transcription factor E2F1 is the principal target of the tumor suppressor pRB. E2F1 promotes cell proliferation by activating growth-promoting genes upon growth stimulation. In contrast, E2F1 contributes to tumor suppression by activating tumor suppressor genes, such as *ARF*, upon loss of pRB function, a major oncogenic change. The transactivation domain of E2F1 has previously been mapped to the C-terminal region. We show here that the N-terminal region of E2F1 is critical for the activation of tumor suppressor genes. Deletion of the N-terminal region dramatically compromised E2F1 activation of tumor suppressor genes. The N-terminal region showed transactivation ability when fused to the DNA-binding domain of GAL4. A search for novel interacting factors with the N-terminal region, using a yeast two-hybrid system, identified the general transcription factor GTF2H2. Overexpression of GTF2H2 enhanced E2F1 activation of tumor suppressor genes and induction of cell death. Conversely, the knockdown of GTF2H2 compromised both. E2F1 binding enhanced the binding of GTF2H2 to target promoters depending on the integrity of the N-terminal region. Taken together, these results suggest that the N-terminal region of E2F1 contains a novel transactivation domain that mediates the activation of tumor suppressor genes, at least in part, by recruiting GTF2H2.

## 1. Introduction

The transcription factor E2F is the principal target of the tumor suppressor pRB [1,2,3]. E2F plays pivotal roles in cell proliferation and tumor suppression in addition to other important biological processes such as apoptosis, differentiation, metabolism, stemness, angiogenesis, and others [4,5,6,7,8,9,10,11,12,13,14,15,16,17,18]. In the resting state of normal cells, the activity of E2F is suppressed by the binding of pRB and its family members p107 and p130 (referred to as RB). Upon growth simulation of cells, RB is inactivated by phosphorylation through cyclin-dependent kinases (CDKs), thereby releasing E2F to activate a group of growth-promoting genes [8,19]. Hence, dysfunction of RB, especially that of pRB caused by oncogenic changes, activates E2F (referred to as deregulated E2F) and results in aberrant cell growth, leading to tumorigenesis. In this context, E2F also activates tumor suppressor genes such as *ARF*, an upstream activator of the tumor suppressor p53 [20], to protect cells from tumorigenesis. Among eight E2F family members, E2F1 is the most potent activator of pro-apoptotic genes [21], thereby playing a central role in tumor suppression [22,23]. Importantly, E2F activity induced by growth stimulation does not activate tumor suppressor genes [24], allowing cell proliferation upon physiologic cell growth stimulation. Moreover, activation of the *ARF* tumor suppressor gene by overexpressed E2F1 does not depend on the heterodimeric partner DP [25], in contrast to activation of growth-related E2F target genes, which is strictly dependent on DP [25,26]. Accordingly, the knockdown of DP results in cell cycle arrest, similar to cellular senescence, in cancer cell lines, even those lacking pRB and p53 functions [27]. These observations indicate that E2F1 activity targeting tumor suppressor genes is distinct from that which activates growth-related genes. Thus, it is important to elucidate the regulatory mechanism of deregulated E2F1 activity in order to understand the molecular basis of tumor suppression that antagonizes the loss of pRB function. However, details of how such E2F1 activity is regulated are not known.

A transactivation domain of E2F1 has been mapped to the C-terminal region [28,29]. However, the N-terminal region of E2F1 shows relatively low homology between other activator E2Fs (E2F2 and E2F3a) compared to the DNA-binding domain and transactivation domain, suggesting the possibility that it has a novel function for E2F1. During our analysis of the regulation of E2F1 activity, we found that deletion of the N-terminal region of E2F1, which is outside of the DNA-binding domain and the previously identified transactivation domain, dramatically compromised the ability of E2F1 to activate tumor suppressor genes. This suggests that the N-terminal region of E2F1 contributes to the activation of tumor suppressor genes possibly through interacting with other factors. We thus explored novel interacting factors of E2F1 by yeast two-hybrid screening using the N-terminal region of E2F1 as the bait. Among the identified candidates, we found the general transcription factor GTF2H2. This raised the possibility that the N-terminal region of E2F1 mediates the activation of tumor suppressor genes through interaction with basic transcription machinery through GTF2H2. We thus examined the roles of the N-terminal region of E2F1 in the regulation of tumor suppressor genes in relation to GTF2H2. Our results show that the N-terminal region of E2F1 contains a novel transactivation domain, which contributes to the activation of tumor suppressor genes, at least in part, by recruiting GTF2H2.

## 2. Materials and Methods

### 2.1. Cell Culture

Human normal fibroblasts (human foreskin fibroblasts: HFFs, obtained from ATCC), human osteosarcoma cell line U-2 OS, and 293A cell line (obtained from Invitrogen, Waltham, WA, USA) were cultured in Dulbecco’s modified Eagle’s medium (DMEM) containing 10% fetal calf serum (FCS).

### 2.2. Plasmid

pARF-Luc(-736), p73(-892)-Luc, pENTR/CMV, pENTR-E2F1, pENTR-12SE1a(∆2–11), and pCMV-β-gal have been previously described [24,30]. The expression vector for N-terminal deleted (∆N) E2F1, in which amino acids 2–83 are deleted, was generated from pENTR-E2F1 by PCR-based mutagenesis. pcDNA3-GAL4DBD expresses the GAL4 DNA-binding domain under the control of the CMV promoter. Expression vectors for the GAL4 DNA-binding domain fused to the N-terminal regions of E2F1 (amino acids 1–83) and E2F3a (amino acids 1–131)—pcDNA3-GAL4DBD-E2F1N and pcDNA3-GAL4DBD-E2F3aN—were generated by cloning nucleotides 141–389 of E2F1 cDNA (NM_005225) and 355–747 of E2F3a cDNA (NM_001949) into pcDNA3-GAL4DBD in-frame, respectively. pG5-Luc is a luciferase reporter plasmid with 5 GAL4 binding sites upstream of adenovirus major late promoter (Promega, Madison, WI, USA). pENTR-GTF2H2 was generated by cloning nucleotide 82–1832 of GTF2H2 cDNA (NM_001515) into *Eco*RI and *Hin*dIII sites of pENTR-CMV. pGBKT7 expresses a protein of interest fused to amino acids 1–147 of the GAL4 DNA-binding domain (DBD) driven by the constitutive ADH1 promoter and has the TRP1 nutritional marker for selection in yeast (Clontech, Palo Alto, CA, USA). pGBKT7-E2F1N was generated by cloning nucleotide 141–392 of E2F1 cDNA (NM_005225), which codes for 1–84 a.a. of E2F1, in-frame with GAL4 DBD. pACT expresses a protein of interest fused to a GAL4 activation domain (AD; amino acids 768–881) driven by the constitutively active ADH1 promoter and has the LEU2 nutritional marker for selection in yeast (Clontech, Palo Alto, CA, USA). The GAL4 AD fusion contains an N-terminal SV40 nuclear localization signal (NLS) that targets the protein to the yeast nucleus. The prey library was a human lymphocyte cDNA library constructed in pACT (Clontech, Palo Alto, CA, USA).

### 2.3. Luciferase Assay

Cells were transfected with reporter and effector plasmids using PEI Max (Polysciences, Warrington, PA, USA). To control for transfection efficiency, a CMV promoter-driven β-galactosidase expression vector, pCMV-β-gal, was included as an internal control. Luciferase activities were measured using the Luciferase Assay System (Promega, Madison, WI, USA) and were normalized to that of β-galactosidase activities. All assays were repeated at least three times and values are shown as means ± SD.

### 2.4. Quantitative Reverse Transcription (qRT)-PCR Analysis

Total RNA was extracted using ISOGEN II (Nippon Gene, Tokyo, Japan). First-strand cDNA was synthesized using PrimeScript 1st strand cDNA Synthesis Kit (Takara Bio, Kusatsu, Japan). Quantitative PCR was performed using PowerUp SYBR Green Master Mix (Applied Biosystems, Waltham, MA, USA) and QuantStudio 3 (Applied Biosystems, Waltham, MA, USA). The primer sets for *CDC6*, *ARF*, *BIM*, *ASPP1*, and *GAPDH* have been described previously [31]. The following primer set was used for *GTF2H2*:

Fw: 5′-GTATGGGATTTCCTCAGCACACC-3′;

Rv: 5′-GGGGAGCAGACACCAAAGTAAGA-3′.

∆∆Ct was calculated for each reaction using *GAPDH* as an internal control. Expression levels of mRNA were normalized to that of *GAPDH* as an internal control and presented as relative mRNA levels or fold induction by E2F1.

### 2.5. Immunoblot Analysis

For protein extraction, the cells were suspended in 5 times the cell pellet volume of RIPA buffer containing a complete protease inhibitor cocktail (Roche, Basel, Switzerland). The mixture was left on ice for 30 min, and the supernatant was collected after centrifugation. The protein concentration was measured using a Protein Assay Kit (BIO-RAD, Hercules, CA, USA). The same amounts of protein samples (30~50 μg) were electrophoresed on an SDS polyacrylamide gel at 100 V for 110 min. Separated proteins were transferred to a PVDF membrane Immobilon (Merck Millipore, Darmstadt, Germany) using a TRANS-BLOT SD SEMI-DRY TRANSFER CELL (BIO-RAD, Hercules, CA, USA) at 12 mA for 60 min. The membrane was blocked using 5% skim milk (BD Biosciences, Sparks, NV, USA) in TTBS (0.1% Tween in TBS) for 1 h and reacted with a first antibody in 0.5% skim milk in TTBS overnight at 4 °C. The membrane was washed 3 times for 20 min with TTBS and reacted with a secondary antibody for 1 h at room temperature. The antibodies used were anti-E2F1 (sc-251, Santa Cruz Biotechnology, Danvers, MA, USA, 1:500), anti-β-galactosidase (AHP1292GA, Bio-Rad, 1:500), anti-GAL4 (DBD) (sc-510, Santa Cruz Biotechnology, Danvers, MA, USA, 1:500), anti-GTF2H2 (HPA047001, Merck Sigma-Aldrich, Darmstadt, Germany, 1:250), and anti-β-actin (A1978, Merck Sigma-Aldrich, Darmstadt, Germany, 1:2000). The secondary antibodies used were anti-mouse IgG-HRP (Jackson ImmunoResearch, West Grove, PA, USA, 1:1000) and anti-rabbit IgG-HRP (NA934, Amersham, Amersham, UK, 1:5000). The signals were detected using LAS4000 (GE Healthcare, Chicago, IL, USA) after treatment with ImmunoStar LD (Fujifilm, Tokyo, Japan) and quantified using ImageJ Version 1.51s (NIH, Bethesda, MD, USA). Western blot original images can be found in Supplementary Materials.

### 2.6. Recombinant Adenovirus

Ad-Con, Ad-E2F1, and Ad-12SE1a(Δ2–11) have been described previously [30]. Ad-ΔNE2F1 was generated from pENTR-ΔNE2F1 using the Vira Power Adenoviral expression system (Thermo Fisher Scientific, Waltham, MA, USA) according to the supplier’s protocol. Infection with recombinant adenoviruses proceeded as previously described [32]. The titer of purified viruses was determined by serial dilution and infection of 293A cells followed by staining the *E2* gene product with a specific antibody raised against synthetic peptides in rabbits (a kind gift from Dr. M Ikeda, Tokyo Medical and Dental University, Tokyo, Japan) and FITC-labeled anti-rabbit IgGs (ab6717, abcam, Cambridge, UK, 1:500).

### 2.7. Immunofluorescence Staining

HFFs were split (1:4) into Nunc Lab-Tek II Chamber Slides (Thermo Scientific, Waltham, MA, USA). The cells were infected with Ad-Con, Ad-E2F1, or Ad-ΔNE2F1 at MOI 200 in 500 µL of DMEM for 1 h. The cells were further cultured for 24 h and fixed with 4% paraformaldehyde in PBS. The cells were permeabilized by 0.2% TritonX-100 in PBS and blocked by 1% bovine serum albumin in PBS. The cells were stained with anti-E2F1 antibody (sc-251, Santa Cruz Biotechnology, Danvers, MA, USA, 1:200) and Alexa Fluor 488 goat anti-mouse IgG (Thermo Fisher Scientific, Waltham, MA, USA, 1:1000) containing 7 μM 4′, 6-diamidino-2-phenylindole (DAPI). The cells were observed with a Nikon A1 confocal microscope (Nikon, Tokyo, Japan).

### 2.8. FACS Analysis

Cells were fixed with 70% ethanol and stained with propidium iodide (50 μg/mL) containing RNase (50 μg/mL). DNA content of the cells was analyzed with a FACSCalibur (Becton Dickinson, Franklin Lakes, NJ, USA). All assays were repeated at least three times and percentages of cells in subG1 DNA content are shown as means ± SD.

### 2.9. Chromatin Immunoprecipitation (ChIP) Assay

ChIP assay was carried out as previously described [24]. The gene-specific primer sets for the *ARF*, *TAp73*, *MCM6*, and *GAPDH* genes are listed below. The antibodies used for immunoprecipitating protein–DNA complexes were anti-E2F1 (sc-251X, Santa Cruz Biotechnology, Danvers, MA, USA), anti-GTF2H2 (HPA047001; Merck Sigma-Aldrich, Darmstadt, Germany), and anti-HA (sc-7392, Santa Cruz Biotechnology, Danvers, MA, USA) as a negative control. Input was one-tenth of the lysates.

*ARF*
FW: 5′-GGCGGTAGGCGGGAGGGAGAGGAA-3′
RV: 5′-CGTGAGCCGCGGGATGTGAACCA-3′
*TAp73*
FW: 5′-CGCAGGCGTCGGGCACAGAGTCG-3′
RV: 5′-GAGGGGAGGCGCCGCGGGGAGTAG-3′
*MCM6*
FW: 5′-CGCGGGCCACGGCTACACTGC-3′
RV: 5′-GCTGCCGCCGCGAGGTCCATA-3′
*GAPDH*
FW: 5′-AAAAGCGGGGAGAAAGTAGG-3′
RV: 5′-CTAGCCTCCCGGGTTTCTCT-3′

### 2.10. Yeast Two-Hybrid Screening

Yeast two-hybrid screening was performed using the Matchmaker Gold Yeast Two-Hybrid System (Clontech, Palo Alto, CA, USA) according to the supplier’s protocol. The yeast strain used was AH109, which requires adenine, histidine, tryptophan, and leucine for cell growth. AH109 contains three reporter genes (*ADE2*, *HIS3*, and *lacZ*) independently driven by GAL4 binding sites placed upstream of the TATA box. The bait plasmid was pGBKT7-E2F1N, which expresses the N-terminal region (1–84 a.a.) of E2F1 fused to the GAL4 DNA-binding domain and has the TRP1 nutritional marker for selection. The prey library consists of human lymphocyte cDNAs constructed in pACT, which expresses the GAL4 transactivation domain fused with the human lymphocyte cDNA library and has the LEU2 nutritional marker for selection. Plasmids were transfected into yeast by the Li acetate method. Positive clones were screened using auxotrophy for histidine and adenine, and blue coloring in the presence of X-α-Gal. Plasmid DNA was retrieved from positive clones by the glass beads method. Pseudo-positive clones were eliminated by screening in the absence of pGBKT7-E2F1N.

### 2.11. Mammalian One-Hybrid System

The reporter plasmid was pG5-Luc, which has 5 tandem copies of GAL4 binding sites upstream of adenovirus major late promoter conjugated with a luciferase reporter gene. The effector plasmid was pcDNA3-GAL4DBD-E2F1N, which expresses the N-terminal region (1–84 a.a.) of E2F1 fused to the GAL4 DNA-binding domain. As negative controls, pcDNA3-GAL4DBD and pcDNA3-GAL4DBD-E2F3aN, which expresses the N-terminal region (1–131 a.a.) of E2F3a fused to the GAL4 DNA-binding domain, were used.

### 2.12. Statistical Analysis

All experiments, except immunostaining, were conducted in triplicate. Data are presented as means ± SD. Statistical comparisons were performed using Student’s *t*-test and Bonferroni correction. A *p*-value < 0.05 was considered as significant.

## 3. Results

### 3.1. Deletion of the N-Terminal Region of E2F1 Compromises Transcriptional Activity

**Figure 1:**
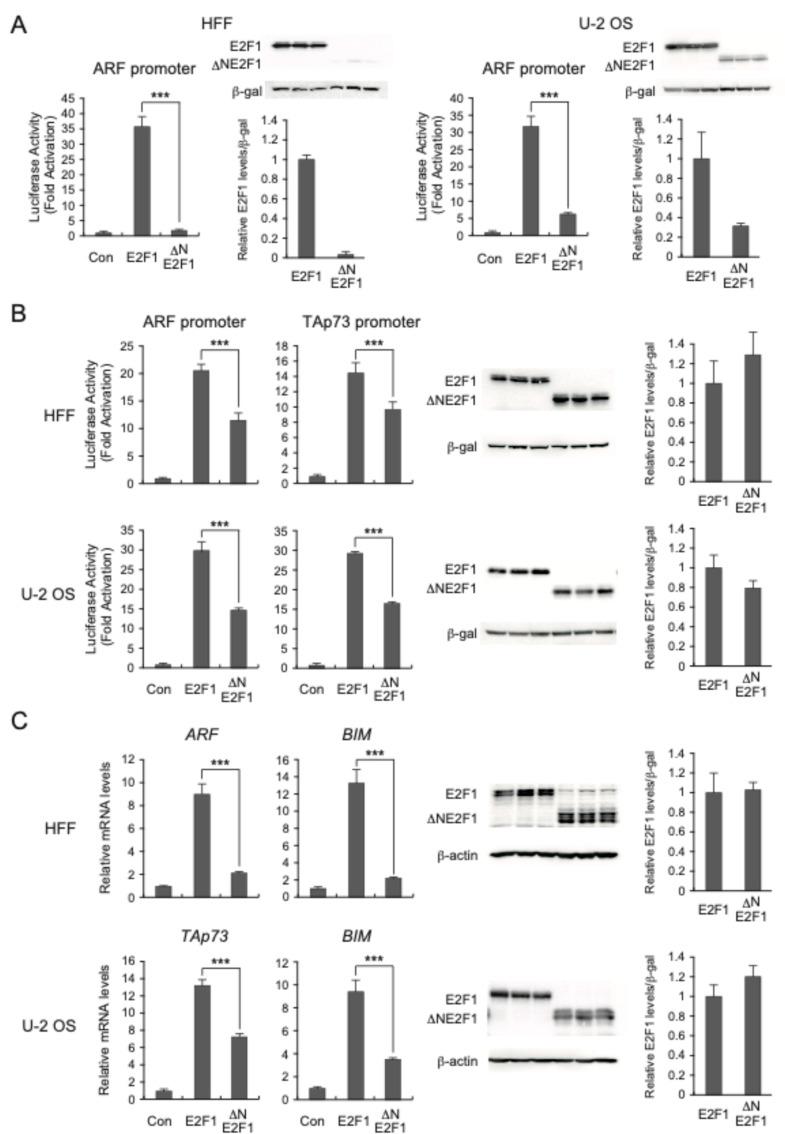
Deletion of the N-terminal region of E2F1 compromises transcriptional activity. (**A**) Deletion of the N-terminal region of E2F1 dramatically compromised transcriptional activity concomitant with reduced protein expression at the same amount of expression vectors. HFFs and U-2 OS cells were split (1:10) into 60 mm dishes. The next day, the cells were transfected with 1.7 µg of pARF (-736)-Luc and 300 ng of pCMV-β-gal as an internal control along with 5 ng of pENTR-E2F1, pENTR-∆NE2F1, or control vector. After 24 h, the cells were washed with PBS, further cultured for 24 h, and harvested. Luciferase activities were measured, normalized by β-galactosidase activity, and presented as fold activation by E2F1. ***: *p* < 0.01. For Western blot analysis, the cells were split (1:10) into 100 mm dishes. The next day, the cells were transfected with 4 µg of pENTR-E2F1 or pENTR-∆NE2F1 and 1 µg of pCMV-β-gal as an internal control. After 24 h, the cells were washed with PBS, further cultured for 24 h, and harvested. Expression levels of E2F1 and ∆NE2F1 were examined by Western blot analysis. β-galactosidase was used as an internal control. Intensities of E2F1 and ∆NE2F1 bands were measured using ImageJ Version 1.51s and adjusted by those of β-galactosidase. Relative expression levels are presented. (**B**) Deletion of the N-terminal region of E2F1 compromised transcriptional activity even at a similar amount of protein expression. HFFs and U-2 OS cells were split (1:10) into 60 mm dishes. The next day, the cells were transfected with 1.7 µg of pARF (-736)-Luc or p73(-892)-Luc and 300 ng of pCMV-β-gal as an internal control along with 5 ng of pENTR-E2F1, 49 ng of pENTR-∆NE2F1, or control vector for HFFs and 5 ng of pENTR-E2F1, 40.5 ng of pENTR-∆NE2F1, or control vector for U-2 OS cells. The total amount of expression vector was adjusted to that of pENTR-∆NE2F1 with the control vector. After 24 h, the cells were washed with PBS, further cultured for 24 h, and harvested. Luciferase activities were adjusted by β-galactosidase activities and presented as fold activation by E2F1. ***: *p* < 0.01. For Western blot analysis, the cells were split (1:10) into 100 mm dishes. The next day, the cells were transfected with 410 ng of pENTR-E2F1 or 4 µg of pENTR-∆NE2F1 for HFFs and 495 ng of pENTR-E2F1 or 4 µg of pENTR-∆NE2F1 for U-2 OS cells and 1 µg of pCMV-β-gal as an internal control. After 24 h, the cells were washed with PBS, further cultured for 24 h, and harvested. Expression levels of E2F1 and ∆NE2F1 were examined by Western blot analysis. β-galactosidase was used as an internal control. Relative expression levels are similarly presented. (**C**) Deletion of the N-terminal region of E2F1 compromised transcriptional activity to induce endogenous target gene expression at a similar amount of protein expression. HFFs and U-2 OS cells were split (1:5) into 60 mm dishes. The next day, the cells were infected with Ad-E2F1 at MOI 100 or Ad-∆NE2F1 at MOI 17 for HFFs and Ad-E2F1 at MOI 5 or Ad-∆NE2F1 at MOI 5 for U-2 OS cells. The cells were further cultured for 24 h and harvested. mRNA levels of *ARF*, *BIM*, and *TAp73* genes were examined by qRT-PCR, normalized by that of *GAPDH*, and presented as relative expression levels. ***: *p* < 0.01. For Western blot analysis, the cells were split (1:5) into 100 mm dishes. The next day, the cells were similarly infected with the viruses. The cells were further cultured for 24 h and harvested. Expression levels of E2F1 and ∆NE2F1 were examined by Western blot analysis. β-actin was used as an internal control. Relative expression levels are similarly presented with β-actin as an internal control.

**Figure 2:**
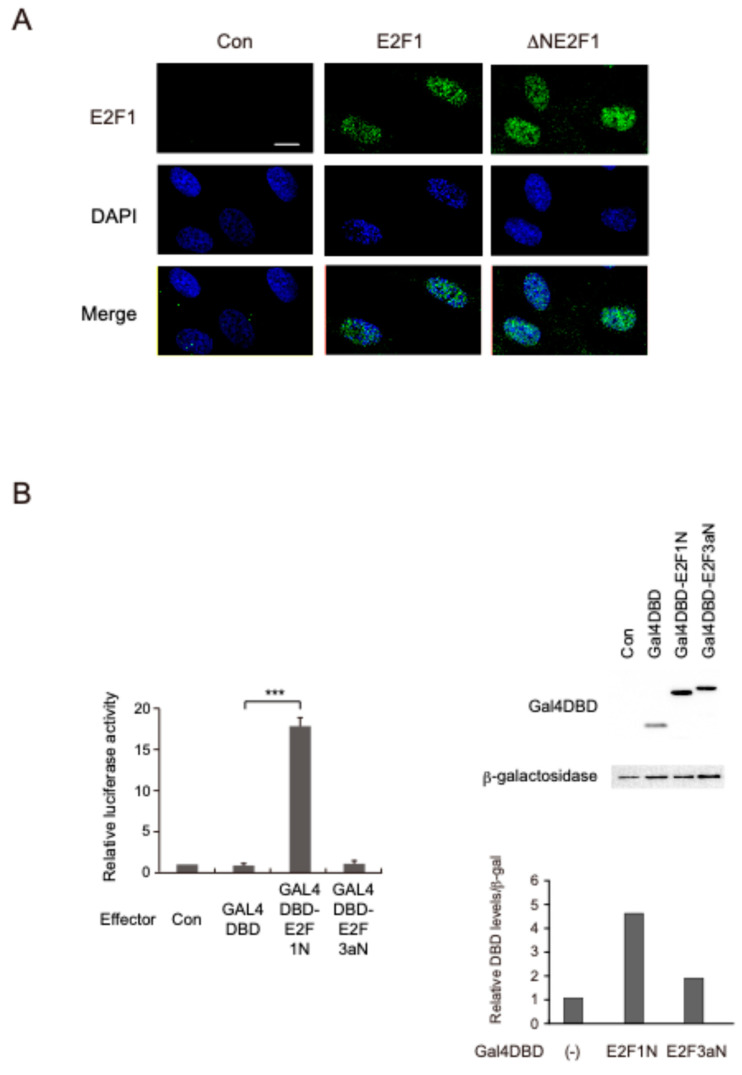
The N-terminal region of E2F1 has transactivation ability. (**A**) Deletion of the N-terminal region of E2F1 does not change the localization of the mutant. HFFs were split (1:4) into Lab-Tek II chamber slides. The cells were infected with recombinant adenoviruses expressing E2F1 or its mutant at MOI 200 and were further cultured for 24 h. The cells were fixed and stained for E2F1 and with DAPI. The scale bar indicates 10 μm. (**B**) The N-terminal region of E2F1 contains a transactivation domain. U-2 OS cells were split (1:5) into 35 mm dishes. After 16 h, the cells were transfected with 0.8 μg of pG5-Luc along with 20 ng of pcDNA3-GAL4DBD or pcDNA3-GAL4DBD-E2F1N and 10 ng of pCMV-β-gal as an internal control. pcDNA3-GAL4DBD-E2F3aN was used as a negative control. The cells were cultured for 24 h and harvested. Luciferase activities were normalized by β-galactosidase activities and are presented as relative luciferase activity. ***: *p* < 0.01. Expression of GAL4DBD effectors was confirmed by Western blot analysis. Intensities of GAL4DBD effectors were measured using ImageJ Version 1.51s and adjusted by those of β-galactosidase. Relative expression levels are presented.

Activator E2Fs (E2F1–E2F3a) show high homology (70–80%) in their DNA-binding, dimerization, and transactivation domains. However, their N-terminal regions exhibit relatively low homology (20–30%), suggesting that the N-terminal region of E2F1 has unique roles for E2F1 functions. Indeed, alignment between the N-terminal region of E2F1 and that of E2F3a, which plays crucial roles in cell proliferation, showed up to only 25.8% homology (Supplementary Figure S1). This observation prompted us to examine whether the N-terminal region of E2F1 has a role in the activation of pro-apoptotic target genes. To assess the functional role of the N-terminal regions of E2F1 in activation of target genes, we generated an expression vector for N-terminal deleted (∆N) E2F1, in which amino acids 2–83 are deleted. To ensure nuclear localization of the mutant, the nuclear localization signal (amino acids 85–91) was retained intact. We first compared the transcriptional ability of ∆NE2F1 and wild-type E2F1, using the same amount of the expression vectors, by reporter assay using ARF promoter in human normal fibroblasts (HFFs). Overexpression of E2F1 in HFFs generates deregulated E2F1 activity, likely due to the expression of an excess of E2F1 relative to endogenous pRB, and activates the ARF promoter. Deletion of the N-terminal region of E2F1 remarkably reduced activation of the ARF promoter by overexpressed E2F1 (Figure 1A, HFF left graph). Deletion of the N-terminal region of E2F1 also reduced activation of the ARF promoter by overexpressed E2F1 in human osteosarcoma U-2 OS cells (Figure 1A, U-2 OS left graph), indicating that the reduction in transcriptional activity upon loss of the N-terminal region is not specific to HFFs. To examine whether deletion of the N-terminal region of E2F1 affected protein expression, we compared expression levels of E2F1 and ∆NE2F1 by Western blot analysis using equivalent amounts of expression vectors. Unexpectedly, deletion of the N-terminal region dramatically reduced E2F1 protein expression in both HFFs and U-2 OS cells (Figure 1A, HFF and U-2 OS right graphs). The reduction in transcriptional activity seemed somewhat comparable to that of protein expression. We thus titrated the number of expression vectors for E2F1 and ∆NE2F1 to obtain equal protein levels by Western blot analysis. To monitor transfection efficiency, the CMV promoter-driven β-galactosidase expression vector was included as an internal control. Band intensities of E2F1 and β-galactosidase were measured by ImageJ Version 1.51s, and E2F1 signals were adjusted by that of β-galactosidase. The ratio of the number of expression vectors for E2F1 and ΔNE2F1 to obtain a similar amount of protein expression (Figure 1B, right panels) was 1:9.8 for HFFs and 1:8.1 for U-2 OS cells. We then performed reporter assays with the ratio of E2F1 and ∆NE2F1 expression vectors that yielded equivalent protein expression. ∆NE2F1, when expressed at equivalent levels, showed significantly reduced transcriptional activity for the ARF promoter in HFFs and U-2 OS cells, compared to wild-type E2F1 (Figure 1B, left graphs), suggesting that the N-terminal region of E2F1 has a role in the activation of target genes. To demonstrate that the reduction in transcriptional activity is not specific to the ARF promoter, we examined the TAp73 promoter, which is also specifically activated by deregulated E2F activity [30]. ∆NE2F1 also showed significantly reduced transcriptional activity for the TAp73 promoter, in HFFs and in U-2 OS cells, compared to wild-type E2F1 (Figure 1B, middle graphs). These results indicate that the N-terminal region of E2F1 contributes to the activation of ARF and TAp73 promoters by E2F1.

To confirm the results of the reporter assays, we next examined whether deletion of the N-terminal region of E2F1 reduces the activation of endogenous E2F target genes. For this purpose, we generated recombinant adenoviruses expressing E2F1 and ∆NE2F1 to allow efficient gene transfer in human normal cells and enable biochemical analyses such as Western blot analysis and qRT-PCR. To obtain a similar amount of protein expression, we titrated the multiplicities of infection (MOI) of E2F1- and ∆NE2F1-expressing viruses in both HFFs and U-2 OS cells and examined expression levels by Western blot analysis. β-actin was used as an internal control. The band intensities of E2F1, ΔNE2F1, and β-actin were measured by ImageJ Version 1.51s, and E2F1 signals were adjusted by that of β-actin. The ratio of MOI for E2F1- and ΔNE2F1-expressing viruses to generate a similar amount of protein expression (Figure 1C, right panels) was 100:17 for HFFs and 5:5 for U-2 OS cells. A lower MOI was required for U-2 OS cells to obtain a similar induction of target genes to HFFs in U-2 OS cells, likely reflecting the higher transduction efficiency of recombinant adenovirus in this cancer cell line. Since basal expression of the *TAp73* gene is undetectable in HFFs, we examined the induction of the *ARF* and *BIM* genes, which are also specifically activated by deregulated E2F activity [31], by overexpression of E2F1 or ΔNE2F1, using qRT-PCR. The induction of both *ARF* and *BIM* genes was dramatically reduced by deletion of the N-terminal region even at a similar level of protein expression (Figure 1C, upper left graphs). The introduction of E2F1 in HFFs induced *ARF* mRNA about 9-fold, compared to only 2-fold by ∆NE2F1 (Figure 1C, upper left graph). A similar reduction in the induction of the *BIM* gene was also observed with ∆NE2F1 (Figure 1C, upper middle graph). Since the *ARF* gene is silenced by DNA methylation in U-2 OS cells, we examined *TAp73* and *BIM* genes in U-2 OS cells. Significant reduction in the induction of the *TAp73* and *BIM* genes was also observed with ∆NE2F1 in U-2 OS cells (Figure 1C, lower left and middle graphs). These results indicate that deletion of the N-terminal region of E2F1 significantly reduces its transcriptional activity.

### 3.2. Deletion of the N-Terminal Region Does Not Compromise Nuclear Localization of ∆NE2F1

To ensure nuclear localization upon deletion of the N-terminal region of E2F1, we kept the nuclear localization signal intact. To confirm the nuclear localization of ∆NE2F1, we examined the cellular localization of E2F1 and ∆NE2F1 by immunostaining in HFFs, after recombinant adenovirus-mediated gene transfer. Both E2F1 and ∆NE2F1 were detected predominantly in the nucleus (Figure 2A), confirming that the effect of deletion of the N-terminal region on transcriptional activity was not due to a change in subcellular localization.

### 3.3. Identification of Novel Interacting Factors with the N-Terminal Region of E2F1

Since the N-terminal region of E2F1 is outside of the DNA-binding domain and transactivation domain of E2F1, we hypothesized that the N-terminal region of E2F1 contributes to the activation of target genes by interacting with other factors. To identify these factors, we explored novel interacting proteins with the N-terminal region of E2F1, using a yeast two-hybrid system with the N-terminal region of E2F1 as the bait. We constructed a yeast expression plasmid in which the N-terminal region of E2F1 was fused to the GAL4 DNA-binding domain and screened it against the human lymphocyte cDNA library fused to the GAL4 transactivation domain as prey. The original screenings of 3.9 × 10^6^ colonies (the library size was 3.4 × 10^6^) identified 50 candidates. To exclude false positives, we re-screened the 50 candidates without the bait and identified 21 candidates as listed in Table 1. Among the candidates, we found the general transcription factor GTF2H2. The main mechanisms of transcriptional activation by transcription factors involve the recruitment of basic transcription factors or chromatin modifiers. Thus, the identification of GTF2H2 as a candidate for interacting factors with the N-terminal region of E2F1 raised the possibility that the N-terminal region of E2F1 interacts with GTF2H2 to contribute to the activation of target genes. We thus focused on GTF2H2 for further analysis.

### 3.4. The N-Terminal Region of E2F1 Contains a Novel Transactivation Domain

The transactivation domain of E2F1 has been mapped to the C-terminal region using CAT assay with GAL4 DNA-binding domain fusions and GAL4 DNA-binding sites [28,29]. Since deletion of the N-terminal region of E2F1 significantly reduced transcriptional ability and we have identified the general transcription factor GTF2H2 as a candidate for novel interacting factors of the N-terminal region of E2F1, we postulated that the N-terminal region may also contain a transactivation domain. We thus examined whether the N-terminal region of E2F1 has transactivation ability using luciferase assay with the GAL4 system in U-2 OS cells. In this assay, the reporter is a luciferase vector, in which the luciferase gene is driven by a minimal TATA box conjugated to five tandem repeats of GAL4 binding sites. The effector was the GAL4 DNA-binding domain (DBD) fused with a protein of interest. Notably, the expression of GAL4 DBD fused with the N-terminal region of E2F1 activated the GAL4 reporter about 18-fold compared to GAL4 DBD alone (Figure 2B). In contrast, fusion of the N-terminal region of E2F3a as a control did not significantly increase transcriptional ability. These results indicate that the N-terminal region of E2F1 contains a novel intrinsic transactivation domain.

### 3.5. Overexpression of GTF2H2 Enhances E2F1 Induction of Target Gene Expression and Cell Death

**Figure 3:**
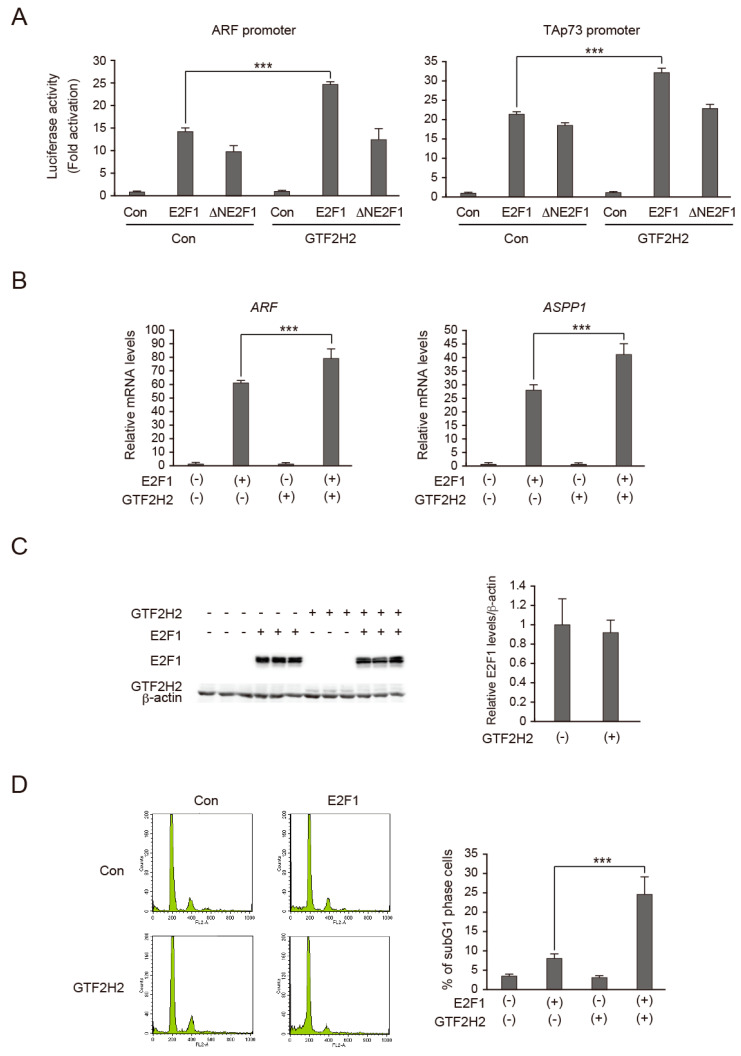
GTF2H2 enhances E2F1-mediated gene expression and induction of cell death. (**A**) GTF2H2 enhances E2F1 activation of ARF and TAp73 promoters. HFFs were split (1:10) into 60 mm dishes. The next day, the cells were transfected with 1.7 µg of pARF(-736)-Luc or p73(-892)-Luc and 300 ng of pCMV-β-gal as an internal control along with 5 ng of pENTR-E2F1, pENTR-∆NE2F1, or control vector and 200 ng of pENTR-GTF2H2 or control vector. After 24 h, the cells were washed with PBS, further cultured for 24 h, and harvested. Luciferase activities were adjusted by β-galactosidase activity and presented as fold activation by E2F1. ***: *p* < 0.01. (**B**) GTF2H2 enhances E2F1 activation of endogenous target gene expression. HFFs were split (1:10) into 60 mm dishes. The next day, the cells were infected with recombinant adenoviruses expressing E2F1 or control virus (MOI 100) along with recombinant adenoviruses expressing GTF2H2 or control virus (MOI 10). The cells were further cultured for 24 h and harvested. Expression levels of the *ARF* (**left**) and *ASPP1* (**right**) genes were examined by qRT-PCR, adjusted by that of *GAPDH* as an internal control, and presented as relative mRNA levels. ***: *p* < 0.01. (**C**) GTF2H2 expression did not affect expression levels of E2F1. Under the same conditions, expression levels of E2F1 were examined by Western blot analysis (**left**). β-actin was used as an internal control. Intensities of the bands were quantified by ImageJ Version 1.51s and relative expression levels of E2F1 adjusted by that of β-actin are presented (**right**). (**D**) GTF2H2 enhances E2F1-induced cell death. Under the same conditions, the cells were further cultured for three days and the percentage of dead cells was examined by FACS analysis based on their subG1 DNA content. The experiment was performed in triplicate and representative data are presented (**left**). The cells in subG1 DNA content were gated and the mean percentage of cells in subG1 is presented (**right**). ***: *p* < 0.01.

Since we identified GTF2H2 as a novel N-terminal region of E2F1 interacting factor and deletion of this region significantly reduced E2F1 transcriptional activity, we examined the effects of GTF2H2 on the activation of target genes by E2F1. We first examined the effect of overexpression of GTF2H2 on activation of the ARF promoter in HFFs, by reporter assay. Expression of E2F1 alone activated the ARF promoter 15-fold, whereas co-expression of GTF2H2 increased activation to 25-fold, indicating that overexpression of GTF2H2 enhanced activation of the ARF promoter by E2F1 (Figure 3A, left panel). In contrast, expression of GTF2H2 did not significantly enhance activation of the ARF promoter by ∆NE2F1, suggesting that enhancement of E2F1 activation of the ARF promoter by GTF2H2 is dependent on the integrity of the N-terminal region of E2F1. Similar results were obtained with the TAp73 promoter reporter construct (Figure 3A, right panel), indicating that the effect is not specific to the ARF promoter.

We also examined whether overexpression of GTF2H2 enhanced E2F1 activation of endogenous target genes in HFFs. For this purpose, we generated recombinant adenovirus expressing GTF2H2. Overexpression of GTF2H2 enhanced E2F1 induction of the ARF gene expression from 60-fold to 80-fold (Figure 3B, left panel). Since the basal level of TAp73 gene expression was under detectable levels, we examined the effect of overexpression of GTF2H2 on the ASPP1 gene, which is also specifically activated by deregulated E2F [31]. Overexpression of GTF2H2 enhanced E2F1 induction of the ASPP1 gene expression from 24-fold to 41-fold (Figure 3B, right panel). To confirm that the enhancement of target gene expression by GTF2H2 was not due to augmentation of E2F1 expression, we examined the expression levels of E2F1 by Western blot analysis (Figure 3C left panel). We measured the signal intensity of E2F1 by ImageJ Version 1.51s and adjusted it by that of β-actin (Figure 3C right panel). The expression levels of E2F1 were not significantly affected by overexpression of GTF2H2. These results suggest that GTF2H2 cooperates with E2F1 to activate tumor suppressor genes.

We next examined whether enhancement of E2F1 induction of pro-apoptotic gene expression by GTF2H2 augments E2F1 induction of cell death. HFFs were infected with recombinant adenovirus expressing E2F1 with or without GTF2H2-expressing virus, further cultured for 3 days, and the percentage of dead cells was examined by FACS analysis based on their subG1 DNA content. Experiments were performed in triplicate and representative data are presented (Figure 3D, left panels). The percentage of cells with subG1 DNA content was measured and means ± SD are presented (Figure 3D, right graph). Co-expression of GTF2H2 significantly enhanced E2F1 induction of cell death.

Taken together, these results indicate that overexpression of GTF2H2 enhances activation of target genes and consequent cell death by overexpressed E2F1.

### 3.6. Knockdown of GTF2H2 Reduces E2F1 Induction of Target Gene Expression and Cell Death

**Figure 4:**
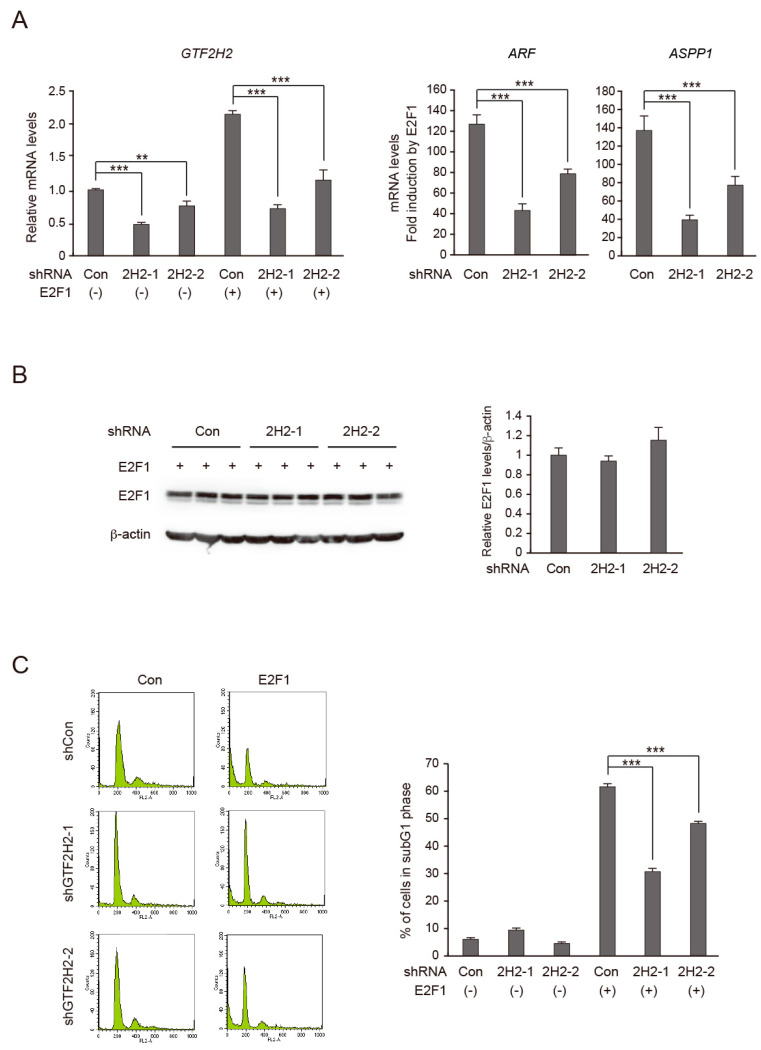
Endogenous GTF2H2 contributes to E2F1-mediated gene expression and induction of cell death. (**A**) Endogenous GTF2H2 contributes to E2F1 activity. HFFs were split (1:15) into 60 mm dishes. The next day, the cells were infected with Ad-shGTF2H2-1, Ad-shGTF2H2-2, or control virus (MOI 20), and further cultured for 2 days. The cells were infected with Ad-E2F1 or control virus (MOI 100), further cultured for 24 h, and harvested. Expression levels of indicated genes were examined by qRT-PCR and normalized by that of *GAPDH* as an internal control. Relative expression levels of GTF2H2 and fold induction of *ARF* and *ASPP1* genes by E2F1 are shown. **: 0.01 ≤ *p* < 0.05, ***: *p* < 0.01. (**B**) Knockdown of GTF2H2 did not significantly affect E2F1 expression levels. Under the same conditions, the levels of E2F1 protein were examined by Western blot analysis (**left**). β-actin was used as an internal control. Intensities of E2F1 bands were measured by ImageJ Version 1.51s, adjusted by that of β-actin, and relative expression levels are shown (**right**). (**C**) Endogenous GTF2H2 contributes to E2F1-induced cell death. Under the same conditions, the cells were further cultured for 4 days and the percentage of dead cells was examined by FACS analysis based on their subG1 DNA content. The experiment was performed in triplicate and representative data are presented (**left**). The cells with subG1 DNA content were gated and the mean percentage of cells in subG1 is presented (**right**). ***: *p* < 0.01.

We next examined whether endogenous GTF2H2 contributes to the activation of target genes by E2F1. For this purpose, we used shRNA-mediated knockdown of GTF2H2 expression, generating recombinant adenoviruses expressing shRNA against GTF2H2 at two different target sequences to exclude off-target effects. Since endogenous GTF2H2 expression was below detectable levels by Western blot analysis (Figure 3C left panel), we examined knockdown effects at the mRNA level by qRT-PCR (Figure 4A left panel). Both shRNAs against GTF2H2 successfully reduced *GTF2H2* mRNA levels. Under the same conditions, the knockdown of GTF2H2 significantly reduced the activation of the *ARF* and *ASPP1* genes by E2F1 (Figure 4A right panels). To confirm that the reduction in activation of the *ARF* and *ASPP1* genes by E2F1 was not due to lower E2F1 expression, we examined levels of E2F1 by Western blot analysis (Figure 4B left panel). E2F1 signal intensities were measured by ImageJ Version 1.51s and adjusted by that of β-actin (Figure 4B right panel). The levels of E2F1 expression were not significantly affected by knockdown of GTF2H2. These results indicate that endogenous GTF2H2 contributes to the activation of the target genes by overexpressed E2F1.

We also examined whether knockdown of GTF2H2 reduced cell death induced by overexpressed E2F1. HFFs were infected with recombinant adenovirus expressing shRNA against GTF2H2, further cultured for 2 days, and infected with E2F1-expressing virus. The cells were further cultured for 4 days and harvested. The fraction of dead cells, based on subG1 DNA content, was examined by FACS analysis. Experiments were performed in triplicate and representative cell cycle profiles are depicted (Figure 4C, left panels). The percentage of cells with subG1 DNA content was measured and presented as means ± SD (Figure 4C, right panel). Knockdown of GTF2H2 significantly reduced E2F1-induced cell death.

Taken together, these results indicate that endogenous GTF2H2 contributes to the activation of target genes and induction of cell death in response to overexpression of E2F1.

### 3.7. E2F1 Recruits GTF2H2 to Target Genes

Overexpression of GTF2H2 enhanced E2F1 activation of target genes and knockdown of GTF2H2 reduced the activation. These results suggest that E2F1 bound to target genes associates with GTF2H2 to facilitate gene expression. We thus explored whether E2F1 binding enhances binding of GTF2H2 to target genes, by chromatin immunoprecipitation (ChIP) assay using anti-E2F1 antibody and anti-GTF2H2 antibody. HFFs were starved of serum, cultured for 2 days, and restimulated with serum to induce physiological E2F1 activity, or infected with recombinant adenovirus expressing E2F1 or adenovirus E1a to generate deregulated E2F1 activity. The cells were further cultured for 1 day and harvested. ChIP assay was performed using anti-E2F1 antibody and anti-GTF2H2 antibody. Association of E2F1 with the promoter region of the *MCM6* gene, a prototypical growth-related E2F target gene [33], was observed not only upon serum stimulation but also in response to overexpression of E2F1 or expression of adenovirus E1a. Association of E2F1 with the promoter region of the *ARF* and *TAp73* genes, tumor suppressor E2F targets [24,30] that specifically respond to deregulated E2F but not to physiological E2F, was not detected in response to serum stimulation but was observed upon overexpression of E2F1 or transduction with adenoviral E1a. Consistent with these results, binding of GTF2H2 to the *MCM6* gene was observed with serum stimulation, overexpression of E2F1, or expression of adenovirus E1a, while the association of GTF2H2 with the *ARF* and *TAp73* genes was detected only in response to overexpression of E2F1 or adenoviral E1a. These results suggest that both physiologically induced E2F1 and deregulated E2F1, associated with target genes, can selectively recruit GTF2H2 to those promoters.

**Figure 5:**
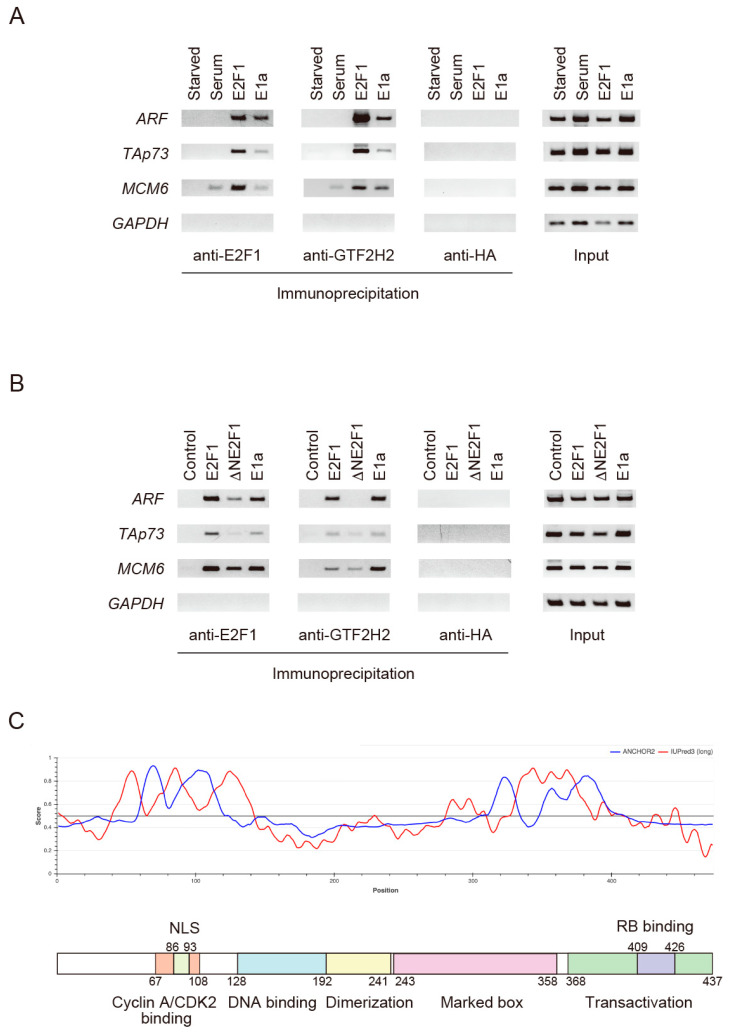
E2F1 recruits GTF2H2 to target promoters. (**A**) The binding of E2F1 enhances that of GTF2H2 to target promoters. HFFs were starved of serum and cultured for 2 days. The cells were re-stimulated with serum or infected with Ad-E2F1 or Ad-12SE1a(∆2–11) at MOI 100, further cultured for 24 h, and harvested. ChIP assay was performed using an anti-E2F1 antibody, anti-GTF2H2 antibody, or anti-HA antibody as an internal control. *ARF* and *TAp73* genes were used as representative of tumor suppressor genes and *MCM6* for that of growth-related genes. *GAPDH* was used as a negative control. The bands show amplified products of PCR reactions against the indicated promoter on the indicated immunoprecipitates, reflecting the binding of E2F1 or GTF2H2 to the promoter. Inputs are amplified products from PCR reactions against the indicated promoter using one-tenth of the lysate employed in immunoprecipitation to show a similar amount of lysate was used for the immunoprecipitation. (**B**) Recruitment of GTF2H2 depends on the integrity of the N-terminal region of E2F1. The ChIP assay was similarly performed, including ∆NE2F1 and without serum stimulation. (**C**) The N-terminal region of E2F1 is disordered. E2F1 amino acid sequences were analyzed using IUPred3 (red line), which predicts intrinsically disordered regions in a given amino acid sequence, and ANCHOR2 (blue line), which predicts the disordered segments that can interact with globular proteins. A region with an IUPred3 score continuously greater than 0.5 indicates a disordered region. A region with ANCHOR2 score continuously greater than 0.5 in a disordered region indicates a disordered segment that can interact with globular proteins.

To examine the roles of the N-terminal region of E2F1 in the recruitment of GTF2H2 to target genes, we next examined whether deletion of the N-terminal region compromised the recruitment of GTF2H2 to target genes under the same conditions. Deletion of the N-terminal region dramatically reduced the binding of GTF2H2 to target genes (Figure 5B). This result supports the notion that the N-terminal region of E2F1 contributes to the activation of target genes by recruiting GTF2H2 to target promoters. Intriguingly, the binding of E2F1 to target promoters was also compromised by the deletion of the N-terminal region of E2F1 (Figure 5B), suggesting that the interaction of E2F1 with GTF2H2 through its N-terminal region stabilizes the binding of E2F1 to target promoters.

### 3.8. The N-Terminal Region of E2F1 Is Disordered

We could not find proteins with significant homology with the N-terminal region of E2F1 by Blast search. Since yeast two-hybrid screening identified 21 candidates, the N-terminal region may be able to interact with other factors in addition to GTF2H2. Accumulating evidence indicates that disordered proteins constitute hubs of signal transduction pathways by interacting with multiple proteins due to unfixed structure and the ability to change conformation according to interacting proteins. We thus analyzed E2F1 amino acid sequences using IUPred3, which predicts intrinsically disordered regions in a given amino acid sequence [34], and ANCHOR2, which predicts the disordered segments that can interact with globular proteins [35], to see if the N-terminal region is disordered. The analysis using IUPred3 showed that the N-terminal region and the marked box region contain disordered regions (Figure 5C). In addition, the predicted disordered region included the segments with large ANCHOR2 scores, suggesting that the disordered segments can interact with globular proteins. These findings support the notion that the N-terminal region of E2F1 is disordered and can interact with different factors according to cellular circumstances.

## 4. Discussion

E2F1 plays pivotal roles in tumor suppression by activating a variety of tumor suppressor genes including *ARF*, an upstream activator of the tumor suppressor p53, upon dysfunction of pRB, a major driver of oncogenesis [20]. Thus, elucidation of the mechanism by which E2F1 exerts selective transcriptional regulation of tumor suppressor genes is crucial to understanding the molecular basis of tumor suppression. The transactivation domain of E2F1 has been mapped to the C-terminal region [28,29]. In this study, we found that the N-terminal region of E2F1 contains a novel intrinsic transactivation domain that recruits the general transcription factor GTF2H2, contributing to the activation of target genes. Deletion of the N-terminal region of E2F1, which is distinct from previously characterized DNA-binding, dimerization, and transactivation domains, significantly reduced transcriptional activity, compared to wildtype E2F1, despite similar protein expression levels (Figure 1B,C). The N-terminal region alone showed transcriptional activity when fused to a GAL4 DNA-binding domain in a mammalian one-hybrid system (Figure 2B). Screening for novel interacting factors with the N-terminal region of E2F1 using a yeast two-hybrid system identified the general transcription factor GTF2H2 (Table 1). Overexpression of GTF2H2 enhanced E2F1 activation of ARF and TAp73 promoters dependent on the integrity of the N-terminal region of E2F1. GTF2H2 also increased E2F1 induction of endogenous target genes and enhanced E2F1-mediated cell death (Figure 3A,B,D). Conversely, knockdown of GTF2H2 reduced E2F1 induction of target gene expression and attenuated E2F1-mediated cell death (Figure 4A,C). Overexpression of E2F1 and expression of adenovirus E1a enhanced binding of GTF2H2 to *ARF* and *TAp73* genes along with enhanced binding of E2F1 in the ChIP assay (Figure 5). Moreover, deletion of the N-terminal region dramatically reduced the recruitment of GTF2H2 to target genes. Taken together, these results indicate that the N-terminal region of E2F1 contains a novel transactivation domain that contributes to the activation of target genes by recruiting GTF2H2.

The decrease in transcriptional activity of E2F1 upon deletion of the N-terminal region is dramatic (Figure 1B,C), considering that the N-terminal deletion mutant retains intact the previously reported transactivation domain in the C-terminal region. The C-terminal transactivation domain has been reported to interact with TATA-binding protein (TBP) and the p62 subunit of THIIH (GTF2H1) [36,37]. Here we showed that the novel transactivation domain in the N-terminal region of E2F1 interacts with the p44 subunit of THIIH (GTF2H2) and possesses intrinsic transactivation ability. These observations suggest that the transactivation domain in the N-terminal region cooperates with that in the previously identified C-terminal region in interacting with TFIIH. This may explain why deletion of the N-terminal region, which leaves the transactivation domain in the C-terminal region intact, dramatically reduced the transcriptional activity of E2F1, and underscores the importance of the novel N-terminal transactivation domain of E2F1 in the activation of the tumor suppressor genes.

Overexpression of GTF2H2 or knockdown of GTF2H2 did not significantly affect the expression of E2F1, which is driven by a constitutively active CMV promoter (Figure 3C and Figure 4B), suggesting that the CMV promoter does not strictly depend on GTF2H2 for promoter activity. In contrast, overexpression of GTF2H2 enhanced E2F1 activation of target genes and knockdown of GTF2H2 reduced E2F1 activation of target genes (Figure 3B and Figure 4A). Moreover, the effects of GTF2H2 on target gene expression paralleled those on induction of cell death mediated by E2F1 (Figure 3D and Figure 4C). These observations point to the roles of GTF2H2 in E2F1 activation of target genes and consequently, induction of cell death, which is crucial for tumor suppression.

The analysis of E2F1 amino acid sequences using IUPred3 and ANCHOR2 showed that the N-terminal region of E2F1 is disordered and included the segment with large ANCHOR2 score, which suggests that the disordered segment can interact with globular proteins (Figure 5C). These findings suggest the possibility that the N-terminal region of E2F1 can interact with different factors according to cellular circumstances. Since yeast two-hybrid screening identified 21 candidates, the N-terminal region may be able to interact with other factors in addition to GTF2H2 that may affect the transcriptional activity of E2F1. The analysis of other candidates is underway.

Deletion of the N-terminal region of E2F1 affected protein expression levels in HFFs and U-2 OS cells. The ratio of expression plasmids to obtain a similar amount of protein expression was 1:9.8 for HFFs and 1:8.1 for U-2 OS cells. From these results, we expected that deletion of the N-terminal region of E2F1 destabilizes the protein. However, the ratio of MOI for E2F1- and ΔNE2F1-expressing virus to generate a similar amount of protein expression was 5.9:1 for HFFs and 1:1 for U-2 OS cells. Thus, the ratio differed between expression plasmids and adenovirus vectors and also between cell lines. Thus, the difference in protein expression levels may not solely be due to protein stability but may also be due to the efficiency of protein expression from expression plasmids and adenovirus vectors, which may differ between cell lines. It is reported that the E2F1 protein is degraded by SCF^SKP2^ and SCF^Cyclin F^ [38,39]. For degradation by SCF^SKP2^, N-terminal 40 amino acid residues are required, which is deleted in our N-terminal deletion mutant. For degradation by SCF^Cyclin F^, the integrity of R90 and L92 is required, which is kept intact in our N-terminal deletion mutant. However, the stability of the protein was not the issue of this study, and we assayed transcriptional activity with a similar amount of protein expression.

The expression level of GTF2H2 protein seems to be extremely low in HFFs since endogenous protein expression was not detected in Western blot analysis even with a relatively high concentration of the antibody (1:250) and even overexpressed GTF2H2 was barely detected (Figure 3C). To examine the physical interaction between E2F1 and GTF2H2, we tried a co-immunoprecipitation experiment. But we could not obtain positive results, likely due to low expression levels of both proteins in HFFs. However, it is highly likely that the N-terminal region of E2F1 interacts with GTF2H2 and contributes to the transactivation of target genes considering the following results: GTF2H2 was identified as an interacting protein of the N-terminal region of E2F1 in yeast two-hybrid screening (Table 1). Overexpression of GTF2H2 enhanced E2F1 activation of ARF and TAp73 promoters depending on the integrity of the N-terminal region (Figure 3A), supporting interaction with the N-terminal region of E2F1. Overexpression or knockdown of GTF2H2 enhanced or reduced E2F1 induction of endogenous *ARF* and *Aspp1* genes, respectively (Figure 3B and Figure 4A). Finally, the binding of GTF2H2 increased upon binding of E2F1 to target promoters in the ChIP assay (Figure 5A), depending on the integrity of the N-terminal region (Figure 5B). These results strongly suggest that E2F1 bound to target promoters recruits GTF2H2 to target genes to activate the genes.

In the ChIP assay (Figure 5B), the binding of ΔNE2F1 to target promoters was reduced compared to wild-type E2F1. This may raise the possibility that the reduced binding of GTF2H2 to target promoters with ΔNE2F1 is due to the reduced binding of ΔNE2F1 to target promoters and is not due to the inability of ΔNE2F1 to recruit GTF2H2. Although this possibility cannot be formally excluded, we do not think this is the case based on the above observations. In addition, since the N-terminal region is outside of the DNA-binding domain of E2F1, deletion of the N-terminal region does not affect the DNA binding ability of E2F1 itself, in theory. We speculate that the interaction of E2F1 with GTF2H2, and consequently with basic transcription machinery, stabilizes the binding of E2F1 to target genes. Hence, reduced binding of ∆NE2F1 to target genes is due to loss of stabilization by basic transcription machinery due to the inability of ΔNE2F1 to bind to GTF2H2.

In almost all cancers, the RB and p53 pathways are disabled [36,40]. The dysfunctional RB pathway generates deregulated E2F1 activity to activate the *ARF* gene, and consequently the p53 pathway [20]. However, the loss of function of the p53 pathway enables cancer cells to survive. Thus, deregulated E2F1 activity, which activates the *ARF* gene, exists specifically in cancer cells [24]. Deregulated E2F1 also activates tumor suppressor genes other than *ARF*, which do not depend on p53 to induce apoptosis, such as *TAp73* and *BIM* [30,31]. Thus, deregulated E2F1 has the potential to induce cell death in p53-disabled cancer cells. Indeed, overexpression of E2F1 in p53-null Saos-2 cells and mouse embryonic fibroblasts induces apoptosis [41,42]. Thus, enhancement of deregulated E2F1 activity in cancer cells is expected to contribute to cancer treatment. Our results point to the crucial roles of GTF2H2 in the activation of tumor suppressor genes by deregulated E2F1, thereby providing a potential new target in cancer treatment.

## 5. Conclusions

The N-terminal region of E2F1 contains a novel intrinsic transactivation domain, which recruits the general transcription factor GTF2H2 to target genes to facilitate gene expression.

## Figures and Tables

**Table 1:** Candidate E2F1 N-terminal region-interacting proteins identified by yeast two-hybrid screening.

Name	Gene ID	Description
ACAT1	38	acetyl-CoA acetyltransferase 1
ACTB	60	actin beta
ACY1	95	aminoacylase 1
CPXM1	56265	carboxypeptidase X, M14 family member 1
DDX5	1655	DEAD-box helicase 5
EEF2	1938	eukaryotic translation elongation factor 2
FAM35A	54537	family with sequence similarity 35 member A
GTF2H2	2966	general transcription factor IIH subunit 2
HSPH1	10808	heat shock protein family H (Hsp110) member 1
IRF5	3663	interferon regulatory factor 5
MAN2B1	4125	mannosidase alpha class 2B member 1
NSUN5	55695	NOP2/Sun RNA methyltransferase 5
PSMB1	5689	proteasome 20S subunit beta 1
RPLP0	6175	ribosomal protein lateral stalk subunit P0
RPSA	3921	ribosomal protein SA
SHARPIN	81858	SHANK-associated RH domain interactor
TIMM50	92609	translocase of inner mitochondrial membrane 50
TSG101	7251	tumor susceptibility 101
WDR1	9948	WD repeat domain 1
WDR77	79084	WD repeat domain 77
ZFP1	162239	ZFP1 zinc finger protein

## Data Availability

The raw data supporting the conclusions of this article will be made available by the authors upon request.

## Supplementary Materials

The following supporting information can be downloaded at https://www.mdpi.com/article/10.3390/biom14111357/s1, Supplementary Figure S1: Alignment of N-terminal region of E2F1 (amino acids 1-83) versus that of E2F3a (amino acids 1-131). Alignment was done using an alignment soft MegAlign of Lasergene (version 13) (DNASTAR). The best match among multiple alignments is shown.
