# Supplementary material for: The N-Terminal Region of the Transcription Factor E2F1 Contains a Novel Transactivation Domain and Recruits General Transcription Factor GTF2H2
Source: Biomolecules. 2024 Oct 25;14(11):1357. doi: 10.3390/biom14111357 (PMC11592155; doi:10.3390/biom14111357)
Supplement: Supplementary file 1 [file biomolecules-14-01357-s001.zip › biomolecules-3231849-supplementary.pdf]

Similarity Index: 25.8

```
               v20       v30       v40       v50       v60       v70
E2F1N    PALEALLGAGALRLLDSSQIVIISAAQDASAPPAPTGPAAPAAGPCDPDLLLFATPQAPRPT
         PALE  L                 A  D  A  A  G AA AA    P             T
E2F3aN   PALEQYLVTAGGGEGAAVVAAAAAASMDKRALLASPGFAAAAAAAAAPGAYIQILTTNTSTT
            ^10       ^20       ^30       ^40       ^50       ^60
```

Supplementary Figure 1. Alignment of N-terminal region of E2F1 (amino acids 1-83) versus that of E2F3a (amino acids 1-131). Alignment was done using an alignment soft MegAlign of Lasergene (DNASTAR). The best match among multiple alignments is shown.
