# Supplementary figures and images for: The N-Terminal Region of the Transcription Factor E2F1 Contains a Novel Transactivation Domain and Recruits General Transcription Factor GTF2H2
Source: Biomolecules. 2024 Oct 25;14(11):1357. doi: 10.3390/biom14111357 (PMC11592155; doi:10.3390/biom14111357)

A

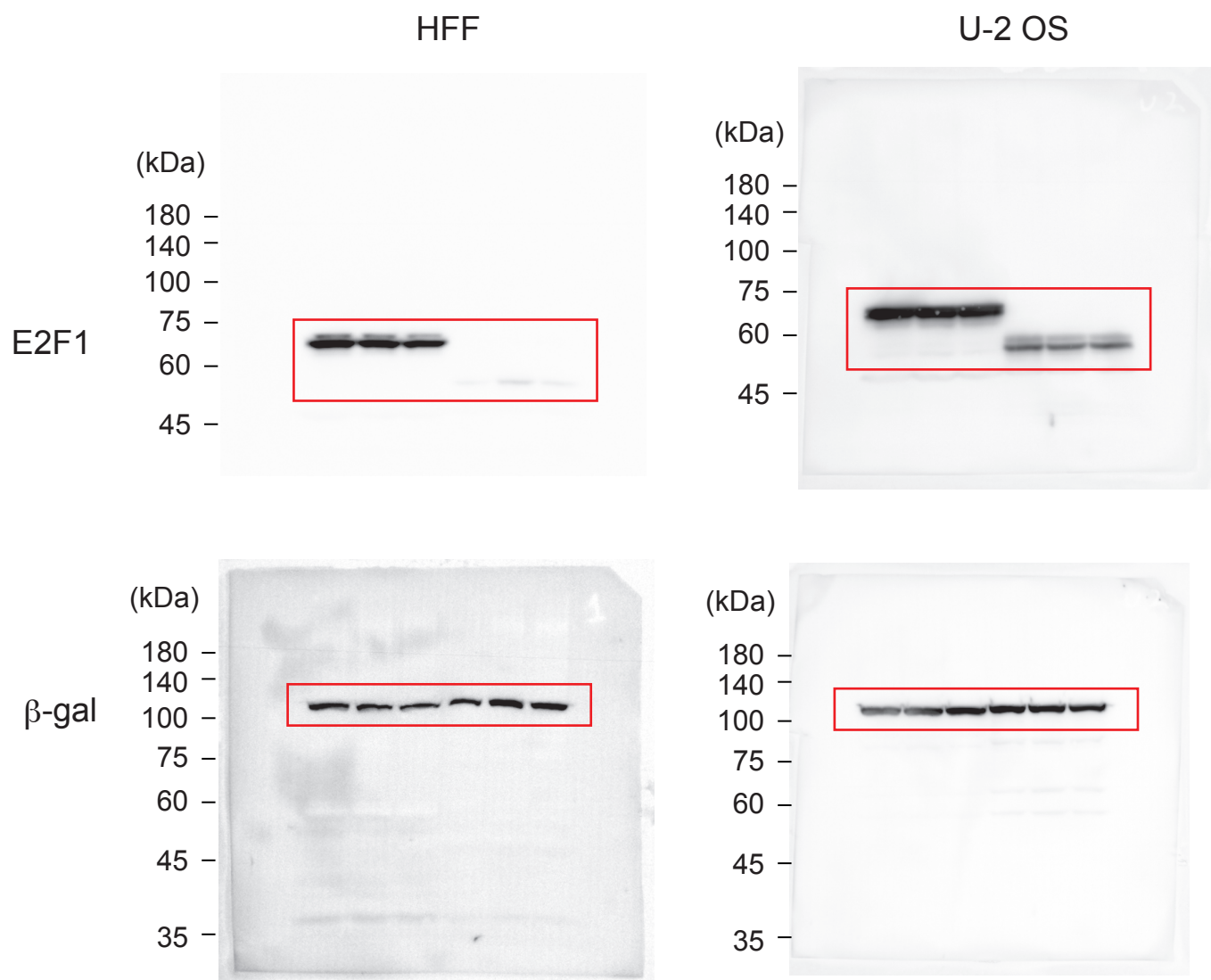

B

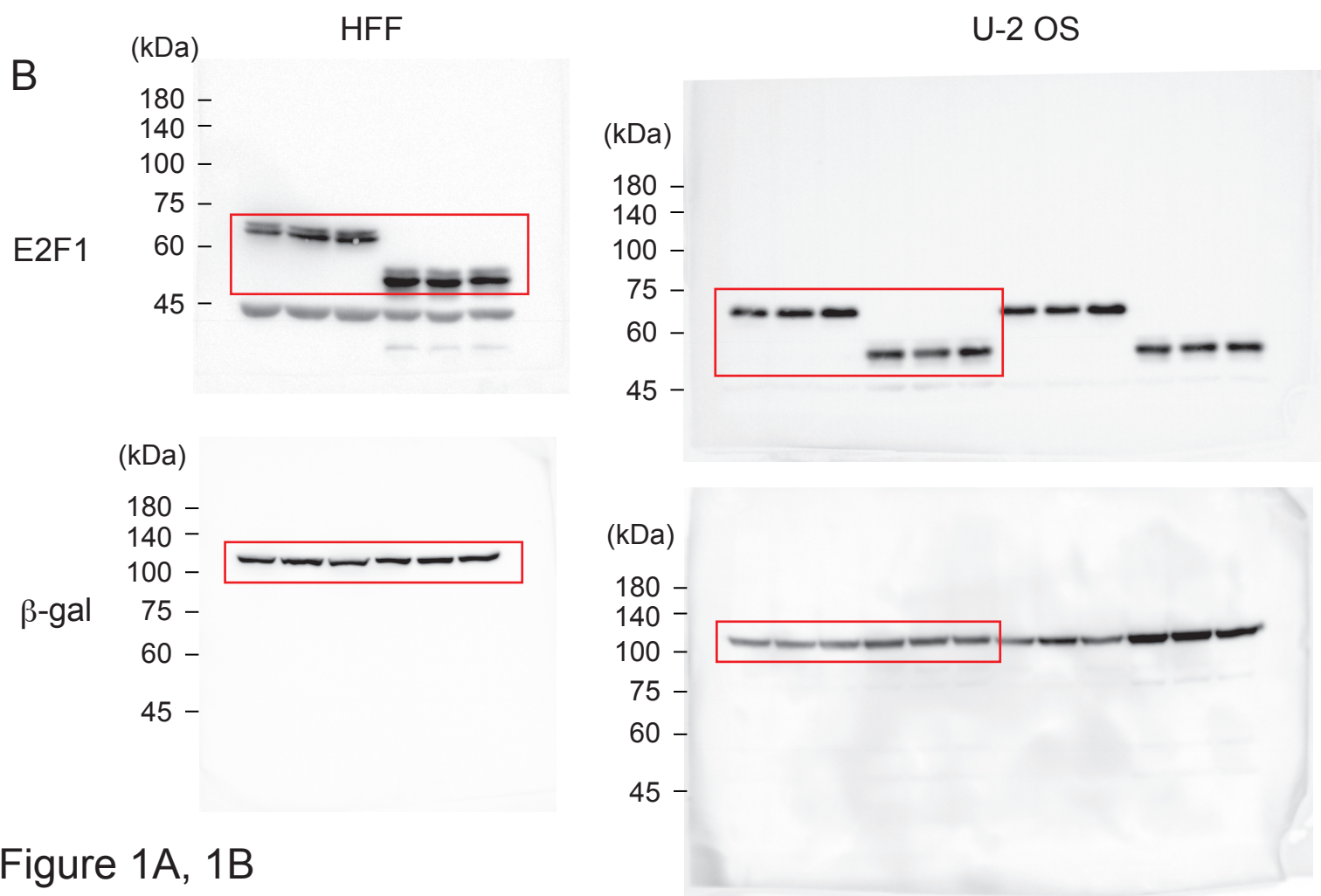

Figure 1A, 1B

Supplement: Supplementary file 1 [file biomolecules-14-01357-s001.zip › Whole Blot Figure 1A, 1B.pdf]

C

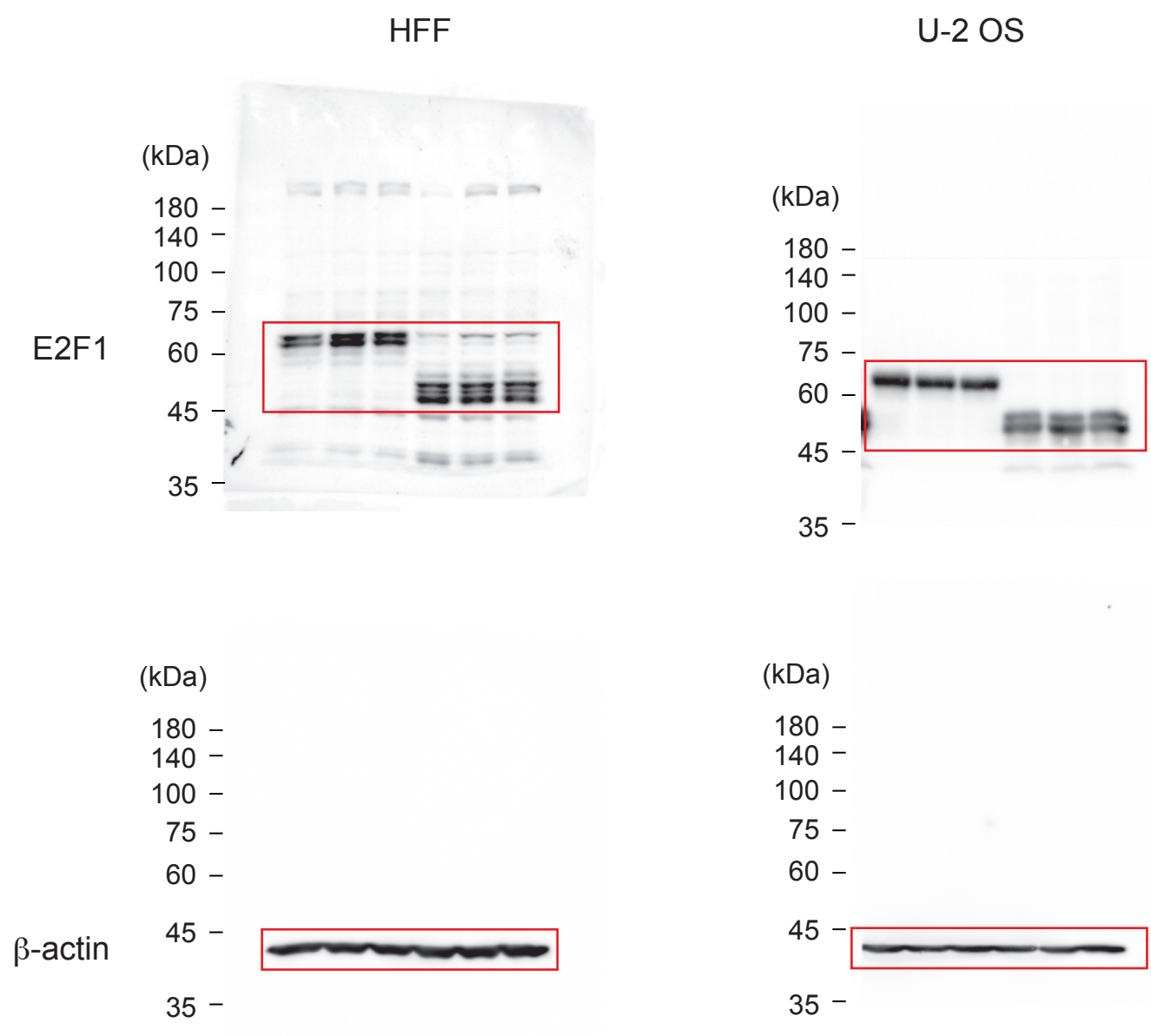

Figure 1C

Supplement: Supplementary file 1 [file biomolecules-14-01357-s001.zip › Whole Blot Figure 1C.pdf]

B

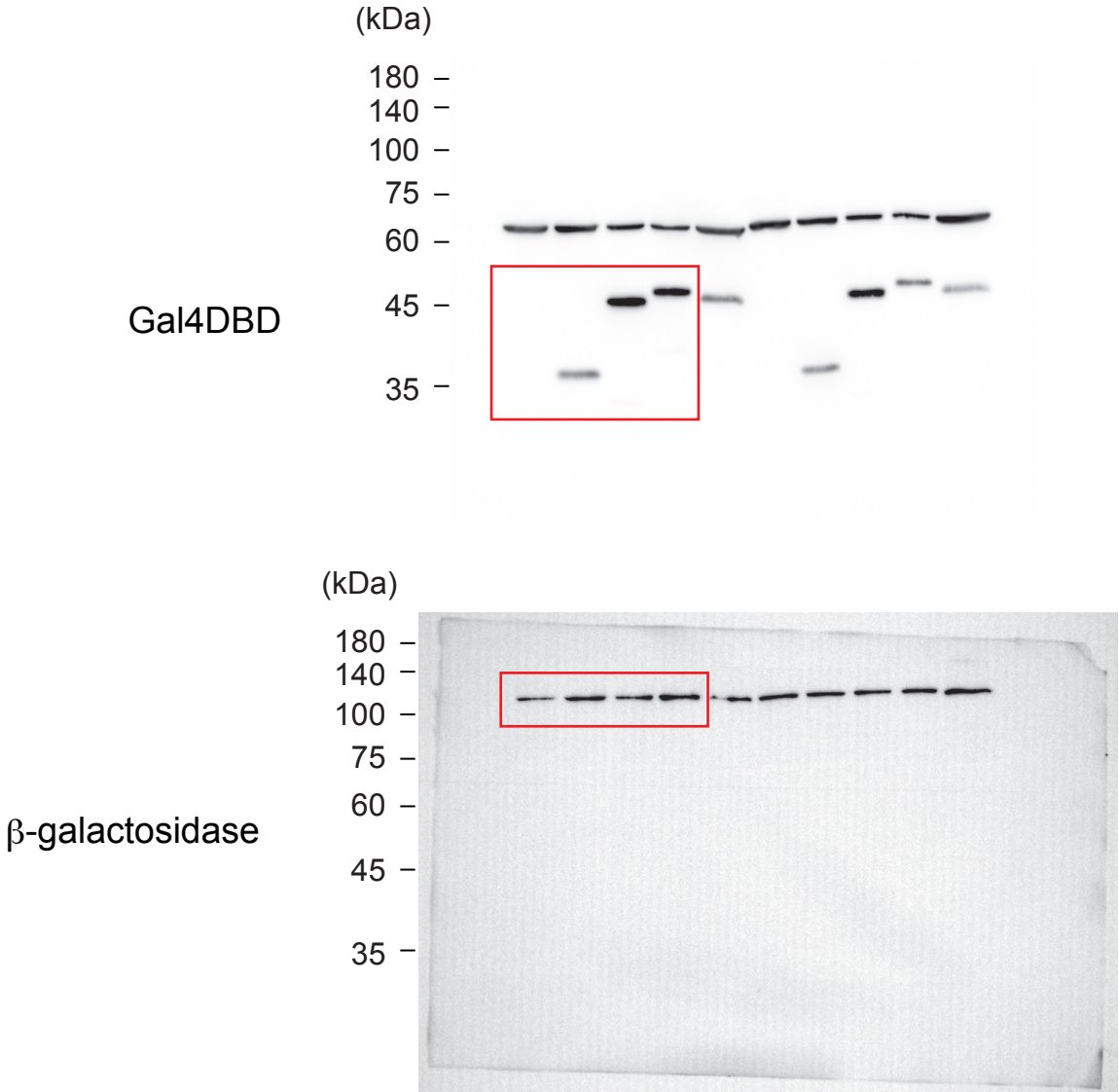

Figure 2B

Supplement: Supplementary file 1 [file biomolecules-14-01357-s001.zip › Whole Blot Figure 2B.pdf]

C

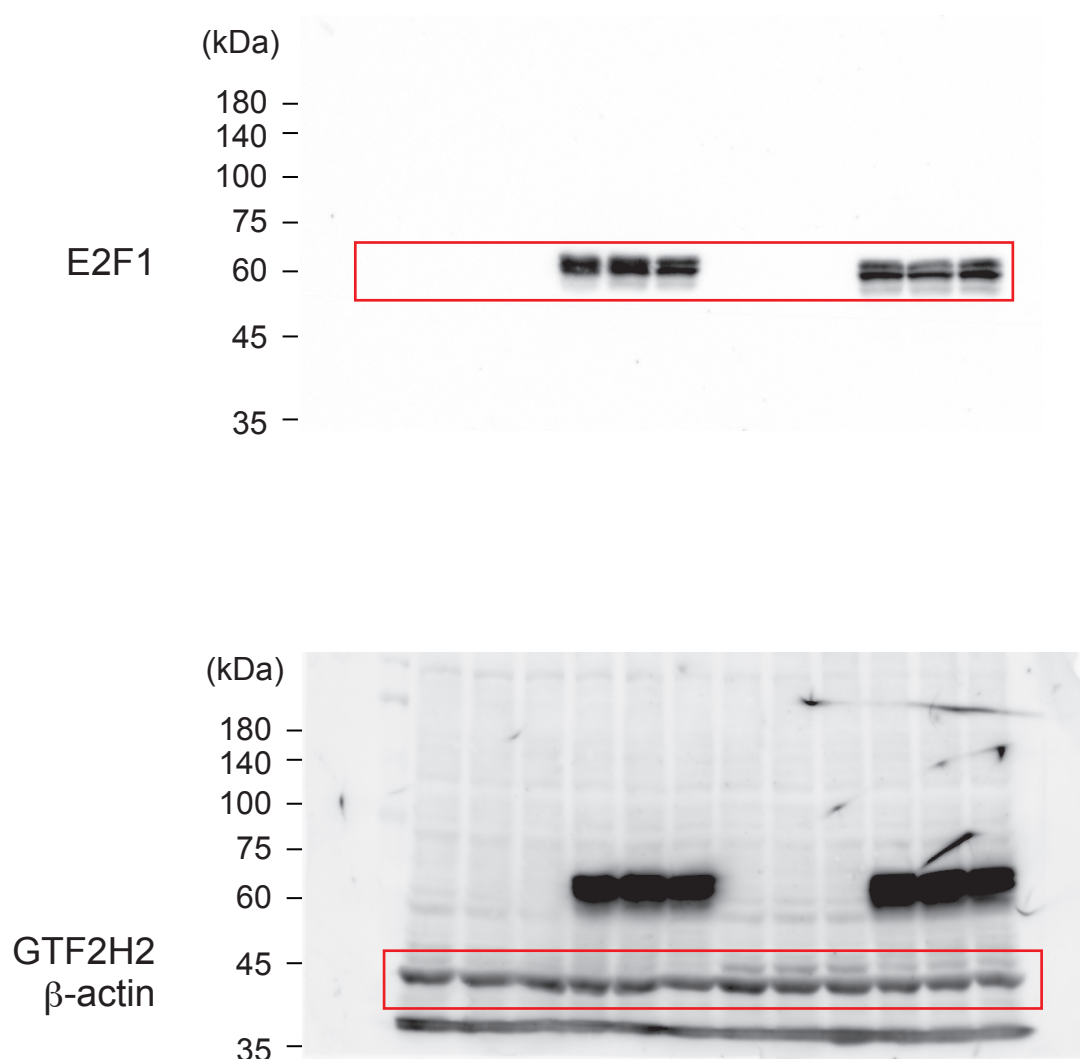

Figure 3C

Supplement: Supplementary file 1 [file biomolecules-14-01357-s001.zip › Whole Blot Figure 3C.pdf]

B

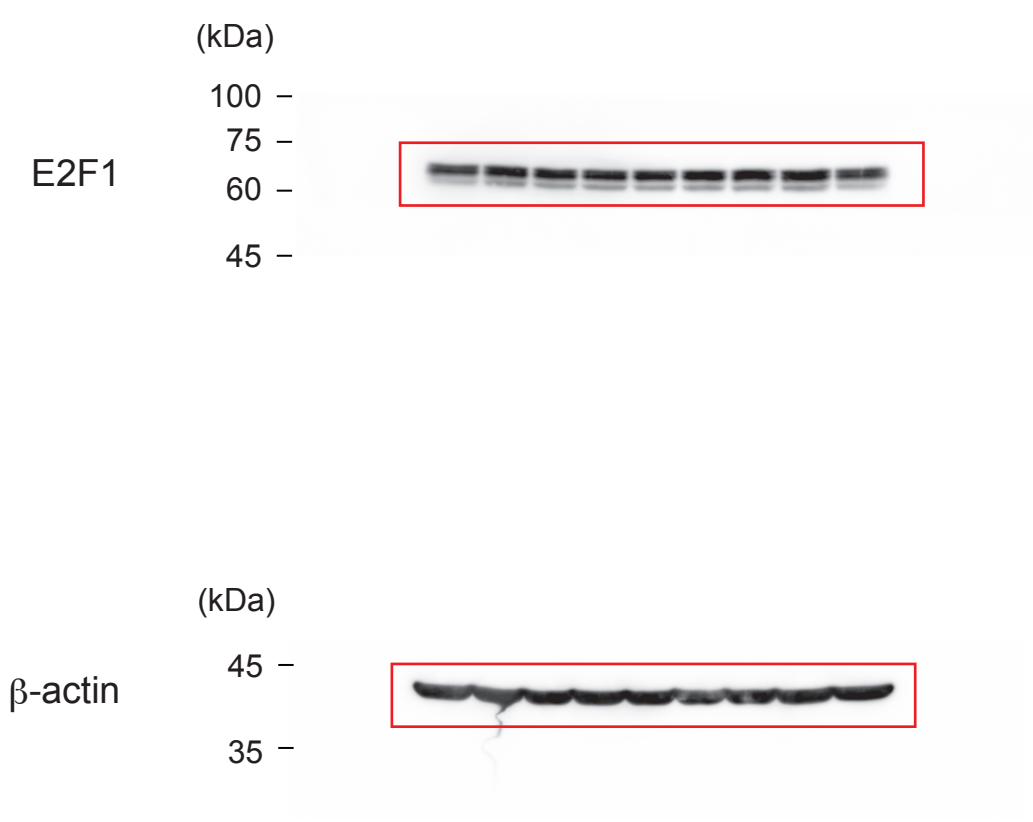

Figure 4B

Supplement: Supplementary file 1 [file biomolecules-14-01357-s001.zip › Whole Blot Figure 4B.pdf]
